# Impact of appetitive and aversive outcomes on brain responses: linking the animal and human literatures

**DOI:** 10.3389/fnsys.2014.00024

**Published:** 2014-03-04

**Authors:** Gregory B. Bissonette, Ronny N. Gentry, Srikanth Padmala, Luiz Pessoa, Matthew R. Roesch

**Affiliations:** Department of Psychology, University of Maryland, College ParkMD, USA

**Keywords:** reward processing, salience, value encoding, appetitive and aversive outcomes, neural encoding

## Abstract

Decision-making is motivated by the possibility of obtaining reward and/or avoiding punishment. Though many have investigated behavior associated with appetitive or aversive outcomes, few have examined behaviors that rely on both. Fewer still have addressed questions related to how anticipated appetitive and aversive outcomes interact to alter neural signals related to expected value, motivation, and salience. Here we review recent rodent, monkey, and human research that address these issues. Further development of this area will be fundamental to understanding the etiology behind human psychiatric diseases and cultivating more effective treatments.

## Introduction

Decision making is a complex process by which an organism must weigh multiple possible outcomes against current and long term goals before deciding on a course of action. Possible outcomes can be grouped into the probability of obtaining something rewarding or avoiding an outcome that is negative or punishing. Although an established body of literature has extensively studied neural systems involved in both of these functions, very few have set out to explicitly study how these neural systems directly reconcile both appetitive and aversive neural signals in a single task. Even fewer have addressed questions related to how anticipated appetitive and aversive outcomes interact to alter neural signals related to expected value, motivation, and salience. Here, we review studies that have addressed this issue in a number of key brain areas, all of which have been shown to exhibit neural activity modulated by expectation of appetitive and aversive stimuli when studied independently. We first review the non-human animal literature and then review studies performed in humans.

We know from a vast literature that neural activity of several regions throughout the brain are modulated by expected outcome, whether it be appetitive or aversive. It is widely assumed that this activity corresponds to an internal representation of how appetitive or aversive that expected outcome is. However, in many cases, change in responses relating to the expectation of emotionally charged outcomes alters other functions related to motivation, salience, arousal and attention that serve to facilitate response mechanisms to approach or avoid.

For example, an association of a particular odor that previously predicted the presence of a predator would be highly salient though lead to a negative association with that odor, while another odor may predict a salient rewarding stimulus, like ripe fruit, but with a positive valence. So while the general idea that appetitive and aversive systems oppose each other in the brain seems logical and useful (Solomon and Corbit, 1974; Daw et al., 2002), stimuli of either valence may drive arousal or enhance attention to stimuli of learned associations (Anderson, 2005; Lang and Davis, 2006; Phelps et al., 2006; Anderson et al., 2011).

**Figure 1:**
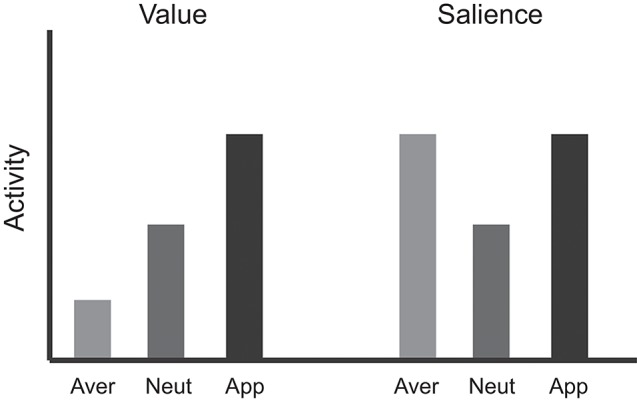
**Logic used to dissociate “value” from other motivational variables**. If activity in a region represents “value” signals, then activation in that region for appetitive stimuli is expected to be greater than aversive stimuli, with responses to neutral somewhere in between. However, if activity represents salience or intensity, activation during both appetitive and aversive stimuli would be greater than that observed to neutral stimuli.

Thus, a key problem is how to dissociate value from these other co-varying factors. A common approach is to vary appetitive and aversive stimuli in the same task. In these types of studies there are typically three basic trial types, such that: (1) a conditioned stimulus (CS) predicts a large reward; (2) a CS predicts a neutral condition or a small (or no) reward; and (3) a CS predicts a small (or no) reward with the threat of an aversive outcome. In animal studies, the aversive outcome may range in quality from time-outs, delivery of a bitter quinine solution, electric shock, or air-puff to the eye (Rolls et al., 1989; Roesch and Olson, 2004; Anstrom et al., 2009; Brischoux et al., 2009; Matsumoto and Hikosaka, 2009; Roesch et al., 2010a; Lammel et al., 2011; Bissonette et al., 2013). In human studies, the aversive outcome may be loss of money, an unpleasant liquid, shock, or an unpleasant odor (Delgado et al., 2000; Anderson et al., 2003; Small et al., 2003; Cooper and Knutson, 2008; Carter et al., 2009; Litt et al., 2011; Choi et al., 2013). If neurons encode *value*, activity should show a decreasing relationship during appetitive, neutral, and aversive trials (Figure 1). If appetitive and aversive stimuli are encoded by independent populations, then neurons should be modulated by either appetitive or aversive stimuli but not both. Finally, if activity is modulated by factors that vary with the strength of appetitive and aversive stimuli, neurons should respond with the same “sign” for appetitive and aversive trials compared to neutral trials (Figure 1). Although the relationship between neuronal responses and blood-oxygenation-related activity obtained via functional MRI is complex (Goense and Logothetis, 2008), it is typically assumed that the same pattern of activity applies to them both—thus, the same type of relationships should be observed at the levels of voxels or regions.

Below we first focus on animal studies that have used similar paradigms to try to dissociate value from motivation, salience, arousal and intensity. We then turn to functional MRI studies in humans.

## Orbitofrontal cortex (OFC)

Orbitofrontal cortex (OFC) encodes expectations about future appetitive and aversive outcomes that are critical for guiding learning and decision-making (Schoenbaum et al., 1998; Roesch and Olson, 2004; Schoenbaum and Roesch, 2005; Plassmann et al., 2010; Morrison et al., 2011; Morrison and Salzman, 2011). For example, neurons in OFC are modulated by cues that predict different appetitive outcomes, such as different food stuffs and magnitudes of reward; other OFC neurons signal when an aversive stimulus is anticipated, such as quinine or air-puff. However, since motivation and value were hard to disentangle in most of these experiments, it was unknown whether neural signals genuinely represented the value of the predicted outcome, or the motivational level associated with obtaining reward or avoiding aversive outcomes. For example, neurons in OFC fire strongly when monkeys anticipate a desirable outcome (Schoenbaum et al., 1998, 1999), but if that outcome is paired with a chance for another, more preferable outcome (Wallis and Miller, 2003), or is devalued through satiation (Rolls et al., 1989), then the rate of firing decreases. This activity modulation might reflect the decrease in value, but it might also reflect changes in motivation. A similar situation holds true for OFC neurons that predict aversive outcomes; activity might reflect how aversive the stimulus is or how motivated the animal is to avoid it.

**Figure 2:**
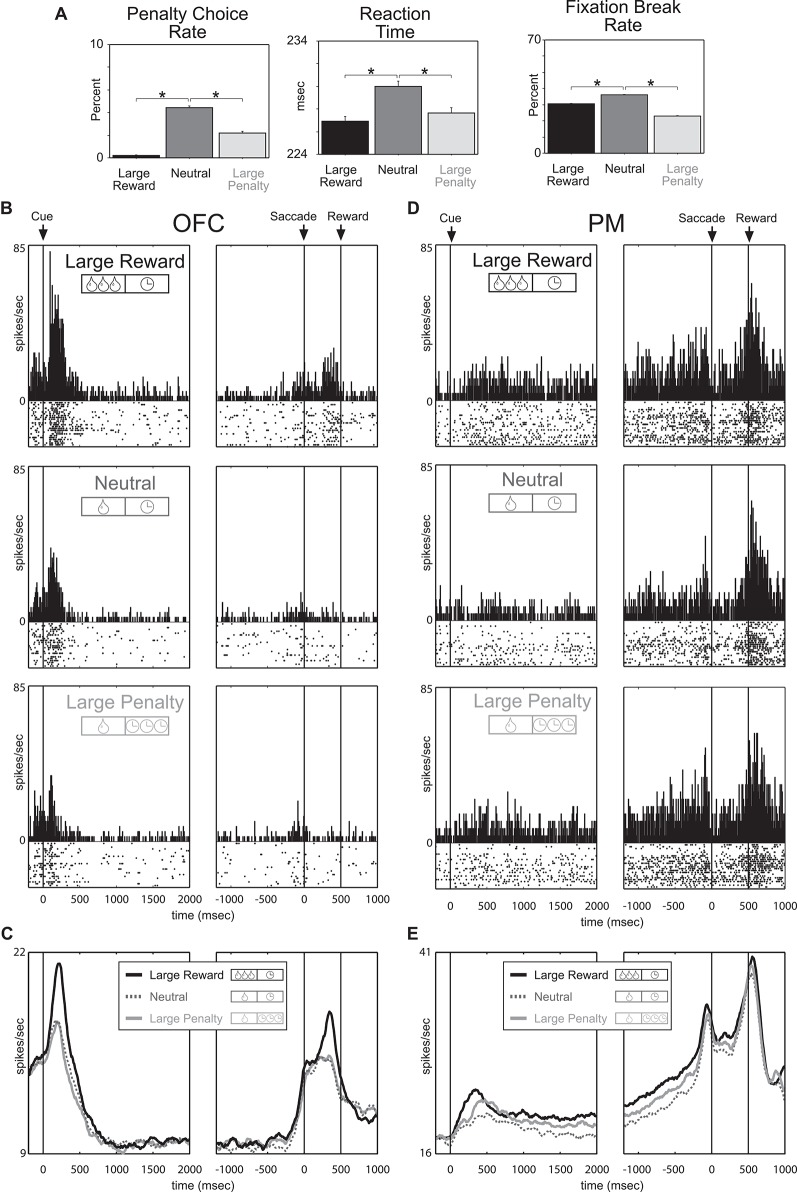
**Premotor and Orbitofrontal cortex encode motivation and value, respectively**. Trials fell into three categories defined by reward-penalty combination: large reward (large reward and small penalty), neutral (small reward and small penalty), and large penalty (small reward and large penalty). **(A)** Performance measures sensitive to reward and penalty size. Penalty choice rate: trials on which the monkeys chose penalty expressed as a fraction of all trials on which they chose reward or penalty. Fixation break rate: trials terminated by a fixation break expressed as a percentage of all trials. Reaction time: average interval between fixation spot offset and saccade initiation on all trials in which the monkey made a saccade in the rewarded direction. Asterisks (all planned comparisons): statistically significant differences at *P* < 0.001. **(B, C)** Neuronal activity in OFC reflects the value conveyed by the incentive cues. **(B)** Shown are data from a single neuron firing during the cue period at a rate that was especially high for large reward and especially low for large penalty. **(C)** Mean firing rate as a function of time under the three incentive conditions for all 176 OFC neurons. **(D, E)** Neuronal activity in premotor (PM) reflects the motivational impact of the incentive cues. **(D)** Shown are data from a single neuron firing throughout the trial at a rate that was high for large reward and large penalty. **(E)** Mean firing rate as a function of time under the three incentive conditions for all 135 PM neurons. Adapted from Roesch and Olson (2004).

To address these issues Roesch and Olson (2004) designed a task to dissociate value from motivation by simultaneously manipulating reward and punishment. The monkeys performed a memory-guided saccade task during which two cues presented at the beginning of each trial indicated the size of the reward the monkey would receive in the event of success (one or three drops of juice) and the size of a penalty that would be incurred in the event of failure (a 1 s or 8 s time-out). Behavioral measures indicated that the monkeys found the large reward appetitive and the punishment aversive; monkeys chose a large reward more often than a small one and avoided a large penalty more often than a small one. More importantly, monkeys were more motivated by large rewards and penalties as compared to smaller ones. Under both the large-reward and large-penalty conditions, the monkeys broke fixation less often, made fewer errors, and were faster to respond, relative to neutral conditions (Figure 2A). Thus dissociation of value and motivation was achieved via simultaneous manipulation of appetitive and aversive outcomes (Roesch and Olson, 2004).

With this task it was predicted that neurons sensitive to the degree of motivation should respond with similar changes in firing rate to increasing the size of either the promised reward or the threatened penalty, thus paralleling the behavior. Indeed, premotor (PM) neurons that are strongly associated with motor output fired continuously during the delay between predictive cues and the behavioral response at a higher rate when either a large reward or a large penalty was expected (Figures 2D, E). Since activity persisted throughout the delay into the time when the monkey was making the behavioral response and because enhancement was observed in neurons with response direction selectivity, changes in firing were interpreted as reflecting motivational enhancement of motor output, as opposed to general arousal or salience. Accordingly, activity in PM reflected the motivational impact of the trial being performed, not its overall value, demonstrating a dissociation between motivation from value at the neural level.

Since OFC is more associated with more emotional/evaluative functions than motor areas, like PM, we expected that OFC neurons would better reflect the value associated with cues and reward delivery. Indeed, in stark contrast to PM neurons, OFC neurons fired most strongly for cues that predicted large reward (with small penalty) and least strongly for cues that predicted large-penalty (with small reward) relative to neutral conditions (small reward and small penalty; Figures 2B–C). Thus the strength of responding in OFC reflected the value conveyed by the combination of reward and penalty cues. Other studies have replicated these results and have further shown that other populations of OFC neurons do not represent the overall value associated with a given situation, but the actual offers being made and the option eventually chosen during performance of a choice task (Padoa-Schioppa and Assad, 2006, 2008; Hosokawa et al., 2007; Morrison et al., 2011). Collectively these studies have shown that OFC has all the signals necessary, at the single unit level, to make reward-guided decisions, as opposed to facilitating behavior through general motivational mechanisms under the influence of predictive appetitive and aversive events.

## Basolateral amygdala (ABL)

Although the mainstream view holds that amygdala is important for acquiring and storing associative information related to both appetitive and aversive outcomes (LeDoux, 2000), there have been hints in the literature that amygdala also supports other functions related to associative learning, such as signaling of attention, uncertainty, and intensity (Saddoris et al., 2005; Belova et al., 2007; Tye and Janak, 2007; Tye et al., 2010; Morrison et al., 2011).

At the single neuron level, basolateral amygdala (ABL) is modulated by the predictability of both appetitive and aversive events, specifically when expectancies are violated (Belova et al., 2007; Roesch et al., 2010a,b; Tye et al., 2010). In other words, ABL neurons increase firing when outcomes are unexpectedly delivered or omitted, events that are highly salient and attention grabbing. We have shown that activity in ABL increases when rewards are unexpectedly delivered (appetitive) or omitted (aversive) in a task in which expected reward varies in size and time to delivery (Roesch et al., 2010a). Others have reported increased activity in ABL when rats were expecting reward, but not delivered during extinction (Tye et al., 2010). In primates, unexpected delivery of appetitive and aversive (air-puff) outcomes during performance of a trace conditioning task with reversals caused amygdala neurons to fire more strongly than when the outcome was totally predictable (Belova et al., 2007). Additionally, it appears that the same populations of ABL neurons which represent appetitive stimuli were also activated by aversive stimuli, regardless of the particular sensory modality from which the experience comes (Shabel and Janak, 2009). This suggests a larger role for ABL in signaling the need for attention in the presence of cues, rather than signaling the associated value of those cues. All of this suggests that amygdala does more than just signal appetitive and aversive stimuli.

Together, these reports suggest that ABL integrates information about appetitive and aversive events and their intensity or salience, possibly in the service of modifying behavior via signaling errors in predictions or recruitment of attentional/executive functions. However, these reports tend to focus on modulation of activity during delivery of appetitive and aversive outcomes. Much less is known about modulation by salience during sampling of cues that predict outcomes. Notably, modulation of amygdala firing for cues that predict appetitive and aversive outcomes appears to occur in separate neurons, suggesting that activity during this period is more related to the valence of the expected outcome. Likewise, cues presented after unexpected events or during response conflict when enhanced attention is necessary do not elicit changes in activity as do errors in reward prediction or commission as observed in other areas, such as anterior cingulate. Thus, ABL might be critical for reporting attentional need, arousal or intensity during sampling of unconditioned stimuli in the service of learning to predict the appetitive and aversive nature of the outcomes during sampling of conditioned stimuli. This is consistent with the finding that ABL interference disrupts development of cue selectivity in other areas, such as OFC and ventral striatum (VS; Hatfield et al., 1996; Schoenbaum et al., 2003a,b; Stalnaker et al., 2007, 2009).

## Ventral striatum (VS)

The connectivity of VS with OFC and ABL suggests that it might also represent the value of expected outcomes. This would be consistent with its proposed role as the “critic” in actor-critic models, where VS generates value predictions about future outcomes, which are used by dopamine (DA) neurons to compute prediction errors (PEs) necessary for updating actions polices in the “actor”, namely dorsal striatum (Houk et al., 1995; Sutton, 1998; Haber et al., 2000; Joel et al., 2002; Redish, 2004; Ikemoto, 2007; Niv and Schoenbaum, 2008; Takahashi et al., 2008; Padoa-Schioppa, 2011; van der Meer and Redish, 2011). However, VS has traditionally been thought to be the “limbic-motor” interface critical for motivating behaviors. Under this framework, one might predict that VS is critical for motivating or facilitating behaviors in response to both appetitive and aversive stimuli—and not for representing value *per se*. Consistent with both of these theories, pharmacological manipulations of VS impact motivated behaviors dependent on value expectations during a variety of tasks (Wadenberg et al., 1990; Berridge and Robinson, 1998; Blokland, 1998; Ikemoto and Panksepp, 1999; Di Ciano et al., 2001; Cardinal et al., 2002a,b; Di Chiara, 2002; Salamone and Correa, 2002; Giertler et al., 2003; Wakabayashi et al., 2004; Yun et al., 2004; Floresco et al., 2008; Gruber et al., 2009; Ghods-Sharifi and Floresco, 2010; Stopper and Floresco, 2011), including reward seeking (Ikemoto and Panksepp, 1999), cost-benefit analysis (Floresco et al., 2008; Stopper and Floresco, 2011), and delay/effort discounting (Cardinal et al., 2001; Ghods-Sharifi and Floresco, 2010).

Motivation is a complex psychological feature, likely arising from assessments of physiological states, understanding and attending to current environmental cues, past reinforcement history, and assessing expected value associated with current contexts. In this light, pharmacological manipulations of the VS will only likely uncover a portion of the story, while single unit recording may uncover separate yet concurrent roles for a brain region, difficult to piece apart with pharmacological work.

Previous single unit work has clearly demonstrated that firing in VS is modulated by the value associated with cues that predict reward in rats (Carelli and Deadwyler, 1994; Setlow et al., 2003; Janak et al., 2004; Nicola et al., 2004; Ito and Doya, 2009; van der Meer and Redish, 2009; Kalenscher et al., 2010; Lansink et al., 2010; van der Meer et al., 2010; Day et al., 2011; Goldstein et al., 2012) and monkeys (Schultz et al., 1992; Shidara and Richmond, 2004; Cromwell et al., 2005; Kim et al., 2009; Nakamura et al., 2012) performing a variety of instrumental tasks, including go/nogo (Schultz et al., 1992; Setlow et al., 2003), lever pressing (Carelli and Deadwyler, 1994; Janak et al., 2004; Shidara and Richmond, 2004; Cromwell et al., 2005; Day et al., 2011), discrimination (Nicola et al., 2004; van der Meer et al., 2010; Goldstein et al., 2012), maze running (van der Meer and Redish, 2009; Kalenscher et al., 2010; Lansink et al., 2010), and eye movement paradigms (Kim et al., 2009; Nakamura et al., 2012). However, from these studies, it was unclear what exactly VS responses represented because none of these studies had independently manipulated value from motivation. To address this issue we adopted a similar behavioral strategy in rats as we did in primates, varying expected reward and punishment so that value and motivation signals could be dissociated (Bissonette et al., 2013).

**Figure 3:**
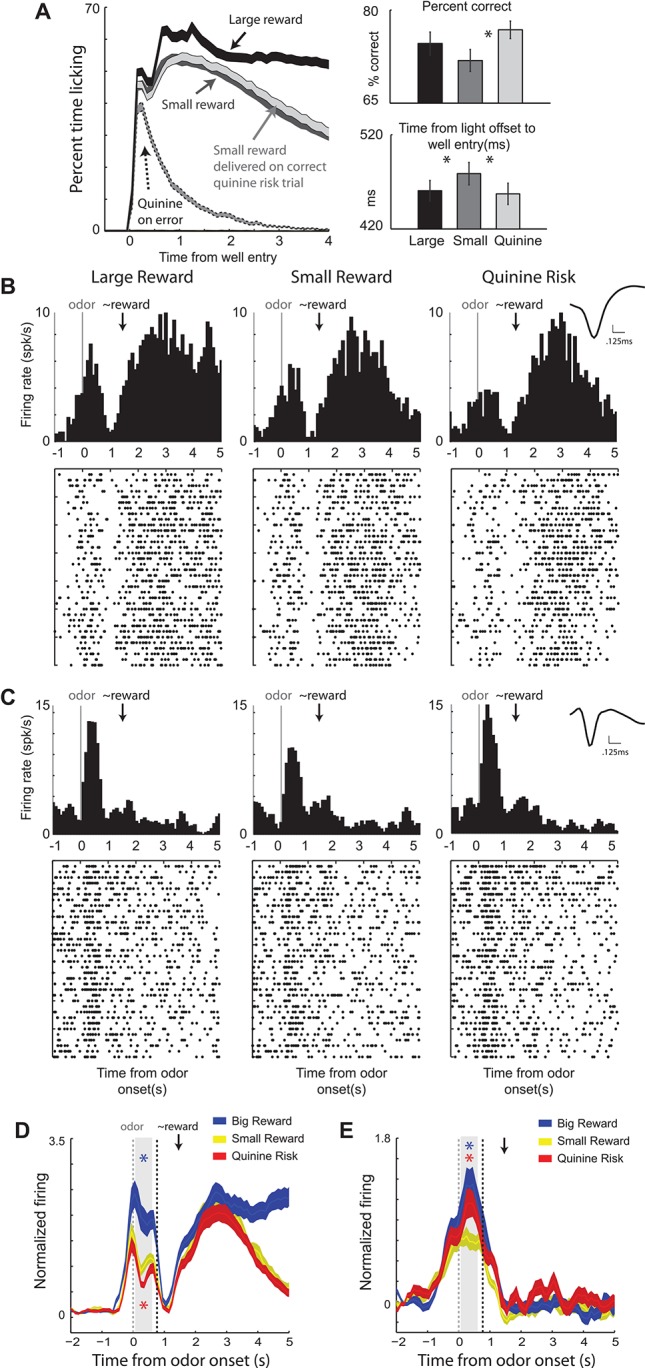
**Ventral striatal neurons encode both value and motivation**. Rats performed a task during which two odors indicated the size (large or small) of the reward to be delivered at the end of the trial. If an error was committed on large and small reward trials, no reward was delivered. A third odor indicated that a small reward would be delivered on correct trials and that quinine would be delivered when rats responded to the wrong well. **(A)** Average lick rate over time during recording sessions. Black = delivery of large reward; Dark gray = delivery of small reward when there was no risk; Light gray = delivery of small reward when there was a risk of quinine. Dashed gray = delivery of quinine on risk trials during which rats went to the wrong fluid well. Average percent correct for the three trial types. Average time taken to move from the odor port to the fluid well in response to the spatial cue lights. **(B–C)** Single cell example of neurons that exhibited firing patterns consistent with value and motivation encoding on correct trials for the 3 trial-types: large reward, small reward, and punishment. Activity is aligned to odor onset (left of dashed box) and reward delivery (right of dashed box). Inset: average waveform (not inverted). **(D–E)** Average normalized firing over all neurons that showed significant increases to both odor cues and reward delivery and those neurons that showed significant increased and decreased firing to cues and rewards, respectively. Firing rates were normalized by subtracting the baseline and dividing by the standard deviation. Ribbons represent standard error of the mean (SEM). Blue asterisks indicate significant differences between average firing during the odor epoch (gray bar) between large reward and small reward trials (blue versus yellow; *t*-test; *p* < 0.05). Red asterisks are the comparison between quinine punishment and small reward trials (red versus yellow; *t*-test; *p* < 0.05). The odor epoch did not include time when lights were on. Gray dashed = onset of odors. Black dashed = earliest possible time lights could turn on. Black arrow marks the average time of reward delivery. Adapted from Bissonette et al. (2013).

Rats were trained on a task in which illumination of a left or right light indicated the location of reward. Prior to the spatial cue, an odor informed the rat of the size of reward and punishment that would result upon correct and incorrect performance, respectively. On two trial-types, there was no risk of punishment, just the potential of a large or small reward for a correct response. On a third trial-type, a small reward was promised for accurate performance, but there was also a risk of punishment (quinine) if the rat performed the task incorrectly. Rats were more accurate and faster to move down to the fluid well in response to the lights on large reward and quinine risk trials compared to small reward trials, demonstrating higher motivation on these trials relative to small reward trials (Figure 3A).

Remarkably, we found that single units in VS encoded both value and motivation. An example of the former is illustrated in Figure 3B. During odor sampling this neuron fired the most and the least for cues that predicted reward and punishment, respectively. This same neuron also fired during delivery of reward, but did not merely encode reward consumption, as evidenced by elevated firing when reward was omitted on error trials (Figure 3D). These results suggest that neurons in VS reflect the expected value of the reward during cue presentation and after the behavioral response. Thus, neurons in VS carry predictive value signals during odor sampling.

This relationship with value was mostly present in the activity of neurons that increased firing to both odor cues and reward delivery. Cue-responsive neurons that showed decreases in firing to reward delivery better reflected the degree of motivation associated conditioned stimuli, as illustrated in Figures 3C, E. For this neuron, activity was stronger for odor cues that predicted large reward and the risk of quinine punishment relative to small reward trials, consistent with representations of enhanced motivation.

Our results suggest that VS fulfills both evaluative and motivational functions, likely via separate neuronal populations, and might be required for integrating both types of information that are central to actor-critic models, as well frameworks that view the VS as a “limbic-motor” interface (Bissonette et al., 2013). All of this work features VS as a common junction point to act, possibly concurrently, to signal value and motivation which leads to the invigoration of particular behavioral actions over others. This idea is consistent with pharmacological studies suggested that DA in the VS had more to do with encoding incentive salience and motivation, rather than evaluative functions (Salamone, 1986; Salamone et al., 1991; McCullough and Salamone, 1992; Salamone, 1994; Koch et al., 2000; Berridge, 2007; Lex and Hauber, 2010; Salamone et al., 2012; Salamone and Correa, 2012; Nunes et al., 2013) and others that show that VS lesions disrupt rats ability to choice between differently valued rewards and to update behavior after devaluation of expected outcomes (Singh et al., 2010; Burton et al., 2013).

## Dopamine

Signals from midbrain DA neurons play a critical role in reinforcement learning by providing a physiological correlate to the well-studied PE. This PE signal guides goal-directed behavior by informing the system which aspects of the environment are appetitive or aversive and initiating actions in order to obtain the good and avoid the bad (Schultz, 1997). Phasic bursts or pauses in neuronal activity, together with resulting neurotransmitter release, encodes this PE signal. The PE signal measures the difference between an expected outcome and the actual outcome in order to inform future behavior. A better-than-expected outcome activates dopaminergic neurons (positive PE) resulting in neurotransmitter release, while a worse-than-expected outcome (negative PE) induces a pause in dopaminergic firing. A fully predictable outcome elicits no change in firing of DA neurons. The same firing pattern applies for sensory cues that come to predict or give information about future rewards. Thus, DA firing and release tends to shift away from the delivery of primary rewards as they come to be predicted by cues during learning, resulting in more or less firing for cues that predict appetitive and aversive outcomes, respectively.

Based on the mismatch of expectation and consequence, the DA signal acts as a teaching mechanism, updating expectations and potential behavioral responses based on feedback received from the environment. DA neurons that fire synchronously and release DA as a result, are reinforced and are more likely to be activated in the future, promoting paired behaviors. The synchronized firing of dopaminergic neurons follows Hebb’s idea that “neurons that fire together, wire together”, but DA must be released in order for reinforcement learning to occur and the synaptic connection between neurons to be strengthened (Montague et al., 1996; Schultz, 1998; Bromberg-Martin et al., 2010). Most of the value signaling described in the brain areas above likely relies on DA to form associations between stimuli and outcomes during learning and decision-making.

Although PE signaling is often studied under paradigms that require animals to approach appetitive stimuli, PE theory holds true for DA signals related to avoiding aversive stimuli, such as air puff and shock. In primates, neurons that encode reward PEs are depressed by unexpected air puff and visual cues that predict them (Bromberg-Martin et al., 2010). Furthermore, DA firing increases when an expected air puff is omitted, an event that is more appetitive or better than expected. A similar story is true in rats performing an instrumental escape-avoidance paradigm. Oleson et al. (2012) showed that phasic DA activity to cue presentation can predict if rats will successfully avoid an upcoming foot shock. Successful avoidance behavior was contingent upon DA release time-locked to the warning light. DA was released at the time of the avoided shock and during cue presentation of successful avoidance trials. Thus, as with appetitive paradigms, DA signals adhere to the general rule of firing more or less strongly for cues and outcomes that are better or worse than expected, respectively (Oleson et al., 2012). Importantly, this signal is dependent on input from OFC (Takahashi et al., 2011).

Notably, not all DA neurons transmit reward PE signals. Other, anatomically discrete, DA neurons appear to be more concerned about the motivational salience of appetitive and aversive stimuli (Matsumoto and Hikosaka, 2009). These DA neurons are triggered by both appetitive and aversive outcomes and the cues that predict them. In experiments where visual stimuli predict either reward or air-puff, these DA neurons fire more strongly for delivery of these outcomes and the cues that predict them, relative to neutral trials where there is no reward or air-puff. Interestingly, these two types of DA neurons, referred to as value and salience encoding neurons, are somewhat segregated in evaluative VTA and SNc, with value encoding cells mostly located in VTA and motivational salience DA neurons in SNc. There exists additional support for the idea that a subset of VTA DA neurons fire preferentially for aversive stimuli in rats, including social defeat, aversive foot shock (Anstrom et al., 2009; Brischoux et al., 2009) and pain inducing plantar injection of formalin to mice (Lammel et al., 2011). Evidence supports the notion that aversive-preferring or salience DA neurons project preferentially to the prefrontal cortex (PFC) and the core of nucleus accumbens (NAc), while reward-preferring or PE DA neurons project preferentially to the ventromedial PFC and the shell of NAc (Bromberg-Martin et al., 2010). Given this data, and the aforementioned idea that these DA neurons may be encoding salience, it seems likely that such a signal would be critical for driving attention/motivation to salient (appetitive or aversive) events promoting learning in regions that these neurons project to, whereas PEs signal might be critical for specifically updating representations of associations between events and their respective outcomes. Thus, DA signals value in the form of PEs, supporting functions related to approach, evaluation and value learning, and also motivational salience, supporting functions related to orienting, attention, and arousal (Bromberg-Martin et al., 2010).

## Parietal cortex and anterior cingulate cortex (ACC)

The most recent debate about value versus salience has focused on the parietal cortex. Parietal neurons have been shown to fire at a rate, dependent on the value of expected actions (action-value) and this signal is critical for making economic decisions about which action produces a better reward. Recently, Leathers and Olson (2012) reported that primate lateral intraparietal (LIP) neurons fire most strongly when a saccade is associated with a large versus small reward. Importantly, they also showed that the same neurons fired more strongly for cues that predicted a large versus small penalty. They suggest that the activity of LIP neurons encode the motivational salience of a cue, rather than the value necessary for decision-making.

In a rebuttal paper, Newsome et al. (2013) suggested that Leathers and Olson (2012) did not replicate delay-period activity as observed in previous experiments, calling into question the population of parietal neurons studied and the ability of the task to tap into these functions that capture action-value (Newsome et al., 2013). Subsequently, Leathers and Olson (2013) replied by pointing out that the key findings of their initial study, namely, that stronger activity was correlated with larger, rather than smaller penalty cues and that neurons signaled salience earlier in the trial during the decision process, were not in question, and that these correlates were found in cells that fired across delays the preceded the response. They suggest that the fact that salience, not value, is encoded by parietal cortex in this task suggests that value encoding is not a general function of parietal cortex. Further work is necessary to determine in what contexts parietal neurons might reflect salience versus value.

Other cortical areas thought to be involved in attention have been recently discussed in the realm of reward-related decision-making and reinforcement learning. Single neurons in macaque ACC show correlates related to unsigned PEs (Hayden et al., 2011), potentially signaling the necessity for additional resources in the face of signaling a need for behavioral modification. Using a variable size/delay task, Bryden et al. (2011) demonstrated rat ACC signaled errors and signaled the need for additional attentional resources during unexpected shifts in value in the same task used to investigate error signing in ABL (Bryden et al., 2011). Unlike activity in ABL, ACC firing was significantly stronger after both unexpected appetitive and aversive events during and before sampling of cues on subsequent trials. This signal likely reflects the salience or attention that is drawn to conditioned stimuli so that contingencies can be updated during learning.

These data are contrasted a bit by work in rhesus monkeys demonstrating ACC encoding of value as it relates to integrating previous outcomes with current choices (Kennerley et al., 2011). Indeed, additional research has suggested that medial PFC (which included parts of ACC) in rhesus monkeys signal both positive and negative PEs of action values (Matsumoto et al., 2007). Others have reported that distinct regions in ventromedial PFC encode rewards and punishments, with ventral and dorsal aspects being more active for appetitive and aversive trial-types, respectively (Monosov and Hikosaka, 2012). The fact that value and salience signals in ACC and parietal cortex appear to go hand in hand are consistent with the need for attentional control to ensure neural processes are prioritized depending on expected events and current behavioral strategy. Indeed neural correlates related to value predictions and spatial attention have been shown to be integrated in clusters of neurons in primate PFC (Kaping et al., 2011). Further research will need to be done to fully separate prefrontal and parietal contributions to signaling value, salience or both using a novel tasks that varies both appetitive and aversive outcomes.

### Human studies

In parallel with the animal literature, human studies have implicated midbrain dopaminergic regions and their projection sites in the striatum and OFC during appetitive processing (Schultz et al., 2000; O’Doherty, 2004; Delgado, 2007; Haber and Knutson, 2010) and regions such as the amygdala and anterior insula during aversive processing (Adolphs and Tranel, 2000; LeDoux, 2000; Craig, 2002, 2009; Davis et al., 2010) Importantly, ventral and dorsal striatal regions are also involved during aversive processing (Jensen et al., 2003; Pruessner et al., 2004; Delgado et al., 2008), while there is some evidence for amygdala and anterior insula activity during appetitive processing (Everitt et al., 2003; Liu et al., 2011). Findings such as these question frameworks that promote appetitive and aversive processing purely in terms of distinct brain regions. Instead, they demonstrate that some of these regions encode factors such as salience and motivational “activation”—not simply value.

Human studies have also attempted to dissociate the processing of value from factors such as salience, intensity, or arousal. These studies have used a wide range of tasks and focused on decision making and PE signals, as well as responses at different task phases, including cue and outcome-related activity. The overall logic used in human studies to attempt to dissociate value from other factors is similar to the one used in the animal literature (Figure 1). As before, three trial types are typically used: (1) appetitive, (2) aversive, and (3) neutral. If activity in a region represents “value” signals, then activation in that region for appetitive stimuli is expected to be greater than aversive stimuli, with responses to neutral somewhere in between. However, if activity represents salience or intensity, activation during both appetitive and aversive stimuli would be greater than that observed to neutral stimuli.

### Decision making

**Figure 4:**
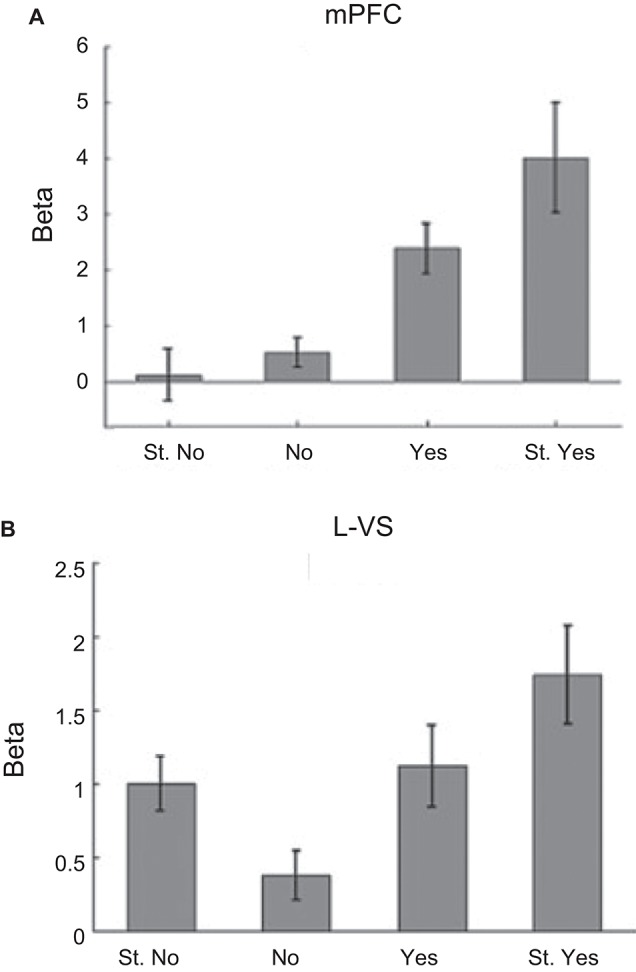
**Dissociation of value and salience signals during a decision-making task**. Human participants were shown pictures of food items that ranged from being highly disliked to highly liked and were asked to make a choice whether or not they would like to eat the item after the experiment (participants in fact consumed these items following scanning). For each picture, participants entered their response on one of the four choices: “Strong No (St. No)”, “No”, “Yes” or “Strong Yes (St. Yes)”. These four types of responses were used to define value and salience signals. The value regressor was defined based on the parametric weights [−2 −1 1 2] and the salience regressor was defined based on the parametric weights [2 1 1 2] corresponding to the four choices above (in that order). **(A)** Evidence for value type signals found in the medial OFC. **(B)** Evidence for both value and salience type signals found in the VS. Adapted from Litt et al. (2011).

Rangel and colleagues used a simple yet elegant decision-making task to disentangle fMRI signals related to value and salience (Litt et al., 2011). Participants were shown pictures of food items that ranged from being highly disliked to highly liked and were asked to make a choice whether or not they would like to eat the item after the experiment (participants in fact consumed these items following scanning). Consistent with previous animal work, value signals were observed in medial OFC (Figure 4A; as well as rostral ACC and PCC). Areas such as dorsal ACC, SMA, and insula generated salience type signals as they produced stronger responses for both “highly disliked” and “highly liked” items. Interestingly, signals in VS exhibited both value and salience type components consistent with the animal literature. As illustrated in Figure 4B, such signals in fact demonstrate that “hybrid” representations that code for both value and salience are also possible (Litt et al., 2011).

### Reward cue processing

Adcock and colleagues utilized a simple cue followed by response task to dissociate value and salience signals in the VS and midbrain (Carter et al., 2009). In the experiment, cues signaled the chance to win monetary rewards (“gain”) or the chance to avoid monetary losses (“loss”) based on fast and accurate performance; baseline conditions involving no gain or loss (“no-gain”/ “no-loss”) were also employed. Cue-related activity in both NAc and VTA increased for both gain and loss trials, thus providing evidence for salience signals in both structures. Furthermore, in both regions, cue-related activity during gain and loss trials was positively correlated across participants providing further evidence for the salience account (Carter et al., 2009).

**Figure 5:**
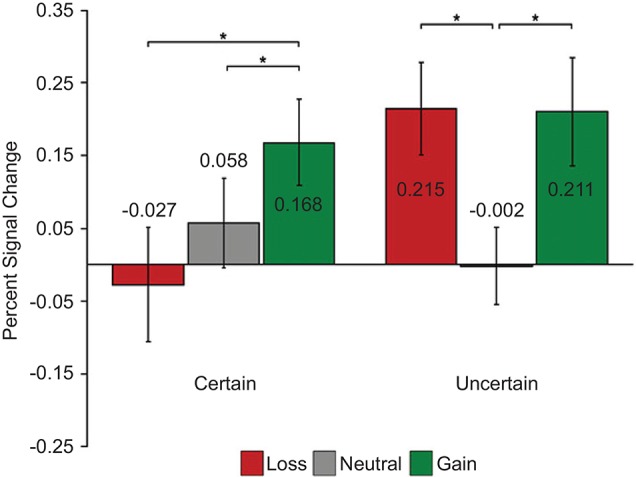
**Dissociation of value and salience signals during a reward processing task with humans**. Each trial started with one of the six cue types: two levels of certainty (“certain”/“uncertain”) crossed with three levels of reward (“gain”/“neutral”/“loss”). After a variable delay period, a visual target appeared and participants pressed a button while the target was on the screen. The duration of the target was adjusted dynamically in each condition separately to maintain approximately 67% task performance. During “gain” trials participants could earn monetary reward; during “loss” trials participants could lose money; during “neutral” trials no win/loss occurred. During “certain” trials, outcomes were independent of performance, whereas during “uncertain” trials outcomes were based on performance. Value signals were found in the NAc when outcomes were “certain” (i.e., independent of performance) and evidence for salience signals were found when outcomes were “uncertain” (i.e., based on performance). Adapted from Cooper and Knutson (2008).

Cooper and Knutson, 2008 also found similar “salience” type responses in the NAc while participants processed cues that signal performance-dependent monetary gains or losses (Cooper and Knutson, 2008; but see Knutson et al., 2001; Breiter et al., 2001). Interestingly, when the outcomes were certain (i.e., independent of performance), they observed increased activity for gain compared to loss cues revealing value type signals in the NAc (Figure 5).

### Reward outcome processing

Delgado and colleagues used a simple card-guessing task to investigate the neural responses related to reward and punishment *feedback* (Delgado et al., 2000). Participants were asked to guess whether the value of the unknown card would be greater or smaller than 5. If they guessed correctly, they received monetary reward; for incorrect guesses monetary punishment was incurred. On neutral trials, where the value of the card turned out to be exactly 5, there was no reward or punishment. They observed value type signals in dorsal and VS during feedback, such that responses were greatest for reward, weaker for neutral and weakest for punishment trials (Delgado et al., 2000). In a follow-up study, they observed that value responses in dorsal striatum were present only when rewards were contingent upon behavior; they were absent when feedback was independent of the behavior (Tricomi et al., 2004).

### Responses to unconditioned stimuli (US)

Another class of experiment has investigated responses to pleasant or unpleasant sensory stimuli themselves. In one case, Anderson and colleagues independently varied the intensity and valence of olfactory stimuli by using pleasant and unpleasant odorants of high and low intensity (Anderson et al., 2003). Responses in amygdala reflected the intensity of the odor, not the valence. In contrast, the OFC revealed value type responses. Specifically, responses in medial OFC were stronger for pleasant compared to unpleasant odors whereas responses in lateral OFC were stronger for unpleasant compared to pleasant odors (Anderson et al., 2003). In a similar study with gustatory stimuli, Parrish and colleagues independently varied the intensity and valence of liquids and found similar evidence for salience signals in the amygdala and value signals in the OFC (Small et al., 2003).

These two studies suggested a general role for the amygdala in the coding of stimulus intensity. Yet, a follow-up study by Dolan and colleagues using olfactory stimuli demonstrated that the activity in the amygdala is best conceptualized in terms of an *interaction* between intensity and valence—that is, an interaction between salience and value (Winston et al., 2005). The authors used high/low concentrations of pleasant/unpleasant/neutral odors and reported that activity in the amygdala was increased for high (versus low) intensity odors *only* when they were pleasant or unpleasant, but not when the odor was neutral. Related valence by intensity interactions have also been observed in the amygdala in the animal literature (Paton et al., 2006).

### Prediction error signals

Several functional MRI studies have used Pavlovian conditioning paradigms to attempt to dissociate value and salience encoding based on the pattern of PE signals.

The logic of these experiments is that regions encoding value would exhibit opposite PE signals for appetitive and aversive stimuli, where a positive PE response would be observed when an appetitive US is delivered or when an aversive US is omitted, and a negative PE response would be observed when an aversive US is delivered or when an appetitive US is omitted. In contrast, regions encoding salience would exhibit similar PE signals for both appetitive and aversive stimuli, where a positive PE would be observed for reinforced outcomes and a negative PE for unreinforced outcomes. Using this logic, Jensen et al. (2007) reported salience type PE signals in the VS, bilateral anterior insula and medial OFC. Similarly, Dreher and colleagues reported salience type PE signals in the striatum (bilateral putamen) and amygdala (as well as anterior insula and ACC) (Metereau and Dreher, 2013). Notably, these studies did not find evidence for *value* type PE signals in the human brain.

#### Salience signals or analysis confound?

**Figure 6:**
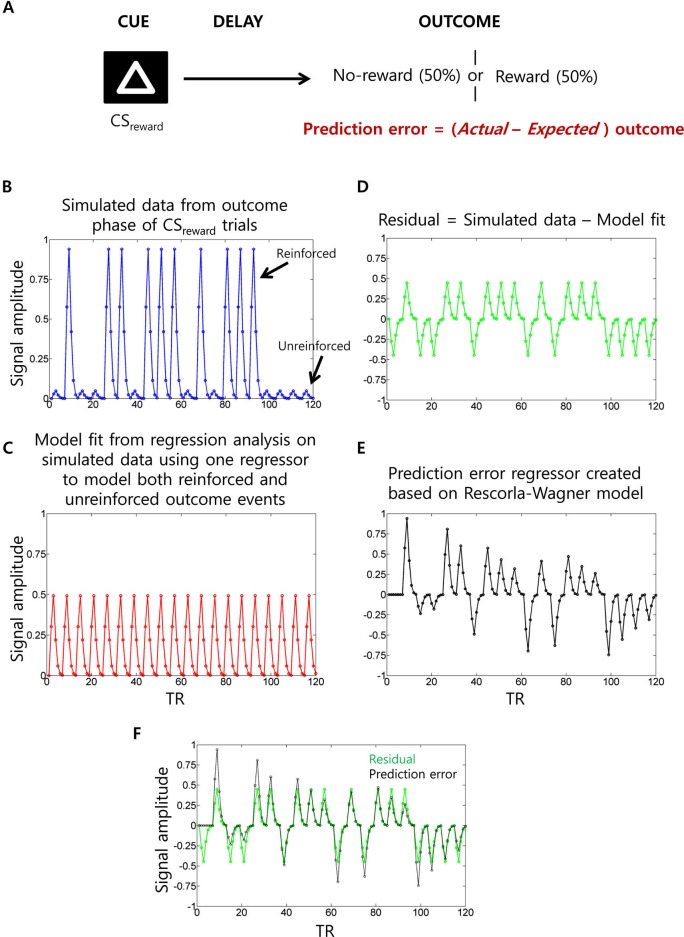
**Prediction error (PE) signal analysis and potential confounds in fMRI analysis. (A)** In a typical appetitive Pavlovian conditioning paradigm, one visual cue (CS_neutral_; not shown) is associated with no-reward (100% probability) whereas a second visual cue (CS_reward_) is associated with 50% probability of receiving reward. PE (i.e., *actual* minus *expected* outcomes) measured at the outcome phase of CS_reward_ trials. **(B)** Simulated fMRI time series data (blue) generated using 10 reinforced and 10 unreinforced outcome events of CS_reward_ trials in a pseudorandom order with 15 s separation between events at a typical TR of 2.5 s. For the sake of simplicity, we have not considered CS_neutral_ trials and the cue phase of CS_reward_ trials (which are typically modeled as separate regressors) and no noise was added to the simulated data. **(C)** When a single outcome phase regressor is used to account for variance during both reinforced and unreinforced outcomes of CS_reward_ trials as typically done, the estimated regression coefficient would be somewhere midway between the activity evoked by reinforced and unreinforced outcomes, as demonstrated by the estimated data fit (red). **(D)** Hence, the residual time series data (green) will show positive values during reinforced outcome events and negative values during unreinforced outcome events. **(E)** Parametric regressor based on trial-by-trial fluctuations of PE values at the outcome phase of CS_reward_ trials calculated using the Rescorla-Wagner rule (Rescorla and Wagner, 1972) (a learning rate of 0.25 was used as often used in fMRI studies). **(F)** The residual time series and the PE regressors are overlaid to show the high correlation between them. Because of this, unaccounted variance during the outcome phase related activity of CS_reward_ trials could be “spuriously” accounted by the PE regressor.

A challenge with functional MRI studies of PEs is that the PE signal is confounded with that of US delivery (Niv, 2009). Specifically, the PE is positive when the US is delivered and negative when the US is withheld. As a consequence, a traditional multiple regression analysis could implicate regions in the generation of PE signals when they are actually responding simply to US delivery. To control for this confound, researchers typically include an additional US regressor (i.e., covariate) for each trial type along with a “parametric” regressor to capture variance related to the PE. Figure 6 illustrates this situation. Unfortunately, this strategy could itself spuriously lead to PE-related activity. For instance, imagine a region that simply responds to the US (e.g., insula activated by electric shock) but has no role in encoding PEs. When a single US regressor tries to account for variance during both reinforced and unreinforced shock outcomes as typically done, the estimated regression coefficient would be somewhere midway between the activity evoked by reinforced and unreinforced outcomes. Hence, the unaccounted variance in this region would have a positive value (i.e., residual) during reinforced outcomes and a negative value (i.e., residual) during unreinforced outcomes. This overall pattern qualitatively matches the shape of the PE regressor. Therefore, one could spuriously detect PE type signals in regions that simply respond to US delivery. Some functional MRI studies have avoided this problem (McClure et al., 2003; D’Ardenne et al., 2008).

### Simultaneous manipulation of appetitive and aversive stimuli

The work that we have discussed so far has considered appetitive and aversive information in isolation. A few recent studies have used stimuli that *simultaneously* incorporate appetitive and aversive information to further understand the role of different brain regions in processing value and/or salience type signals.

In a decision making paradigm, Tobler and colleagues investigated two kinds of salience signals that can only be distinguished in decisions that involve simultaneous costs and benefits (Kahnt and Tobler, 2013). When appetitive and aversive stimuli are presented in *isolation*, salience can be captured by the absolute value of the stimulus (i.e., |App| or |Aver|). But when appetitive and aversive stimuli are presented *simultaneously*, salience could be of two types: one based on the absolute value of the “total” (i.e., |App + Aver|), another based on the sum of the absolute values (i.e., |App| + |Aver|). Tobler and colleagues found evidence for the latter type of salience signal in a site in the temporo-parietal junction (TPJ). Consistent with previous studies, they also found evidence for value based signals in the VS (though they did not detect salience-related signals in the VS). A handful of additional decision making studies have used simultaneous appetitive and aversive stimuli to the same effect (Talmi et al., 2009; Park et al., 2011).

In a recent study, we were also interested in characterizing responses to stimuli containing both appetitive and aversive information. In the study, we investigated the interactions between the anticipation of reward and/or threat (Choi et al., 2013). Participants were presented with four advance cues to alert them of the possibility of: (1) reward/no shock, (2) reward/shock, (3) no reward/no shock, and (4) no reward/shock. Reward was contingent on performance whereas shock was independent of performance. This procedure juxtaposed two competing ideas. One, in line with what we have been discussing, for conditions involving simultaneous reward and threat, enhanced activity would reflect a type of salience signal (given the presence of both dimensions); the other predicted that the presence of both appetitive and aversive stimuli would lead to a “competition” between them. Skin conductance data acquired during scanning demonstrated an interaction between reward and threat processing, such that reward and threat effects were reduced by threat and reward, respectively. In terms of brain responses, several brain areas exhibited this type of reward-threat trade-off, including midbrain, caudate, putamen, and anterior insula.

### Limitations of functional MRI studies

Single unit recordings in midbrain and VS have identified separate populations of neurons coding for value and salience (Matsumoto and Hikosaka, 2009; Bissonette et al., 2013). The coarse spatial resolution of typical functional MRI studies prevents them from measuring separate signals for the separate populations. Indeed, in some cases, the measured fMRI response could be based on the combined activity of underlying value and salience processing neurons. Consider also single-unit studies revealing separate populations of neurons coding for appetitive and aversive stimuli (e.g., Ungless et al., 2004). In such cases, if a region shows salience type fMRI responses, it could be due to the contribution from separate underlying neuronal populations, which would be engaged by appetitive and aversive stimuli. But here it is worthwhile noting that some single-unit studies in humans (Laxton et al., 2013) and monkeys (Amemori and Graybiel, 2012; Monosov and Hikosaka, 2012) have revealed neurons coding for appetitive and aversive stimuli within the *same* population. These studies, together with human studies that revealed the dependence of valence signals on their salience (e.g., during active versus passive task processing) are consistent, in broad terms, with meta-analytic findings reporting little evidence for processing of discrete emotion categories in distinct brain regions (Lindquist et al., 2012).

A second issue is that the sluggish nature of hemodynamic responses makes it challenging to unambiguously disambiguate responses to different task phases, for example, “cue”, “anticipation”, and “outcome” phases. In contrast, the high temporal resolution of electrophysiology provides rich information to investigate the dynamics of value and salience representations (e.g., Matsumoto and Hikosaka, 2009). Importantly, high temporal resolution in single unit studies also allows the investigation of responses at the outcome phase independent from short-latency responses linked to the sensory properties of US (Fiorillo et al., 2013).

## Discussion

In this paper, we reviewed how the brain encodes appetitive and aversive events in both non-human animals and humans. This line of research is important, as understanding the neural processing behind appetitive and aversive stimuli is critical to understand what drives different behavioral responses. Much of the literature has approached these problems by studying how an animal associates a particular odor with a potential predator, or by investigating how a visual cue is associated with a tasty ripe fruit. The behaviors enacted in each of those scenarios would be, naturally, very different (alertly avoid, or boldly engage). However, few situations in real life are as cut and dry. Often, predators prowl near locations and objects that prey animals enjoy (near watering holes, food sources, migratory routes, following the mating calls of animals), and attaining rewards may require dealing with cues that signal aversive events (extracting honey from wild bees, picking fruit from thorny plants).

As reviewed here, despite differences in the species investigated and the techniques utilized, some consensus has started to emerge regarding the encoding of both value and other related motivational signals. Yet, both apparent discrepancies and unresolved issues remain and need to be addressed in future work. The combined evidence reveals that the OFC has a representation of value that is relatively “pure”. The VS carries both value and salience signals that appear to be generated by different neuronal populations. Amygdala responses are modulated by stimulus intensity, though the signal is clearly moderated by the valence (i.e., value) of the stimulus.

**Figure 7:**
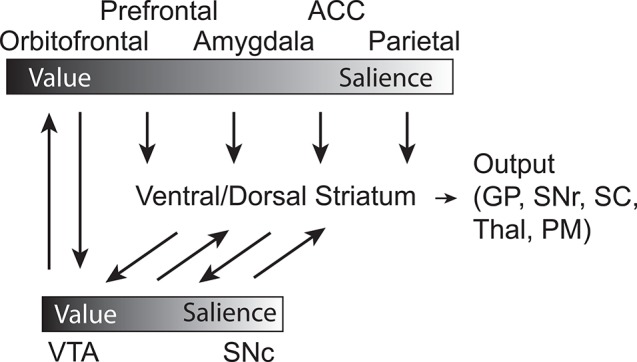
**Circuit diagram demonstrating connectivity between brain regions and their relative location on a sliding scale of value to salience, with the influence of DA signaling integrated**. Gradient bars represent relative encoding of value and salience. Orbitofrontal Cortex—OFC, Prefrontal Cortex—PFC, Basolateral Amygdala—ABL, Anterior Cingulate Cortex—ACC, Parietal Cortex—Parietal, Dorsal Medial Striatum—DMS, Dorsal Lateral Striatum—DLS, Ventral Tegmental Area—VTA, Substantia Nigra compacta—SNc, Superior Colliculus—SC, GP—Globus Pallidus, Thalamus—Thal, Substantia Nigra reticulata—SNr, Premotor Cortex—PM.

Taken together, the work described here suggests a circuit by which OFC represents value expectancies necessary for guiding decision-making and learning. These signals depend on ABL, which not only encodes associative appetitive and aversive information during sampling of conditioned stimuli and across states, but integrates value and intensity/salience during delivery of appetitive and aversive outcomes. OFC and ABL both broadcast this information to VS and DA neurons, which carry both evaluative (VTA) and motivational salience (SNc) signals in separate populations of neurons (Figure 7). PE signals generated by VTA DA neurons provide feed-forward information to more dorsal-medial and dorsal-lateral regions in striatum, which are critical for goal-directed and habitual behaviors, respectively. Parietal and ACC likely increase attentional control to ensure that neural processes are prioritized depending on expected actions and unsigned errors in reward prediction. From this research it is clear that we have to continue to compare and contrast how neural systems reconcile both appetitive and aversive stimuli, and continue to disambiguate the meaning of signals modulated by valence and how they relate to subsequent behavior.

In terms of issues that will drive future research, we can highlight at least three. The first concerns the types of representation in parietal cortex. Are they closer to value based or are they better conceptualized in terms of salience? The second concerns the study of PEs in the human brain with functional MRI. As illustrated, it can be challenging to separate “true” PEs from responses to US delivery. Consequently, it is unclear at the moment if PE signals reflect salience representations across a wider set of regions of the brain as suggested by the human work (e.g., insula, dorsal striatum, amygdala, ACC), or if in some cases they may have resulted from responses to the US itself. This is an area that we believe future work is clearly needed, both non-human work investigating a wider group of regions, and human work that more effectively deals with potential confounds. A third issue is related to functional MRI as a methodology. Both issues of spatial and temporal resolution pose important challenges to being able to investigate value signals in the brain. These clearly need to be addressed more effectively; perhaps with newer techniques that allow finer temporal sampling (every 500 ms or less) of hemodynamic responses and finer spatial resolution (less than 1 mm) can go some way toward mitigating current issues (though higher temporal sampling can only go so far given the low-pass nature of the hemodynamic response). In any case, we anticipate exciting times ahead as the field advances the understanding of how the brain encodes value and other motivational variables.

## Conflict of interest statement

The authors declare that the research was conducted in the absence of any commercial or financial relationships that could be construed as a potential conflict of interest.
